# The Role of Cholesterol on Triterpenoid Saponin-Induced Endolysosomal Escape of a Saporin-Based Immunotoxin

**DOI:** 10.3390/ijms21228734

**Published:** 2020-11-19

**Authors:** Wendy S. Smith, David A. Johnston, Harrison J. Wensley, Suzanne E. Holmes, Sopsamorn U. Flavell, David J. Flavell

**Affiliations:** 1 The Simon Flavell Leukaemia Research Laboratory, Southampton General Hospital, Southampton SO16 6YD, UK; harrison.wensley@googlemail.com (H.J.W.); SuzanneH@leukaemiabusters.org.uk (S.E.H.); BeeF@leukaemiabusters.org.uk (S.U.F.); 2 Biomedical Imaging Unit, University of Southampton Faculty of Medicine, Southampton General Hospital, Southampton SO16 6YD, UK; D.A.Johnston@soton.ac.uk; 3 Clinical and Experimental Sciences, University of Southampton Faculty of Medicine, Southampton General Hospital, Southampton SO16 6YD, UK; 4 Abcam, Cambridge Biomedical Campus, Cambridge CB2 0AX, UK; 5 Faculty of Medicine, University of Southampton, Southampton General Hospital, Southampton SO16 6YD, UK

**Keywords:** saponin, endosomal escape, cholesterol, immunotoxin, augmentation, saporin

## Abstract

Cholesterol seems to play a central role in the augmentation of saporin-based immunotoxin (IT) cytotoxicity by triterpenoid saponins. Endolysosomal escape has been proposed as one mechanism for the saponin-mediated enhancement of targeted toxins. We investigated the effects of lipid depletion followed by repletion on *Saponinum album* (SA)-induced endolysosomal escape of Alexa Fluor labelled saporin and the saporin-based immunotoxin OKT10-SAP, directed against CD38, in Daudi lymphoma cells. Lipid deprived cells showed reduced SA-induced endolysosomal escape at two concentrations of SA, as determined by a flow cytometric method. The repletion of membrane cholesterol by low density lipoprotein (LDL) restored SA-induced endolysosomal escape at a concentration of 5 µg/mL SA but not at 1 µg/mL SA. When LDL was used to restore the cholesterol levels in lipid deprived cells, the SA augmentation of OKT10-SAP cytotoxicity was partially restored at 1 µg/mL SA and fully restored at 5 µg/mL SA. These results suggest that different mechanisms of action might be involved for the two different concentrations of SA and that endosomal escape may not be the main mechanism for the augmentation of saporin IT cytotoxicity by SA at the sub-lytic concentration of 1 µg/mL SA.

## 1. Introduction

Targeted toxins based on ribosome inactivating proteins (RIPs) have been the subject of considerable research but to date their successful clinical use as therapeutic agents has been limited (see [1] for a review). One of the major factors that limits the efficacy of these protein-based conjugates is the efficiency of their internalisation by receptor-mediated endocytosis (RME) and subsequent release from the endolysosomal compartment into the cytosol, where the toxin component can act catalytically on target ribosomes. Poor internalisation of the targeted toxin [2], recycling back to the cell surface after internalisation [3] or trafficking of the toxin moiety to the lysosomes with subsequent degradation [4,5] can all contribute.

Immunotoxins (ITs) are hybrid conjugates consisting of an antibody-based domain, which acts as the target specific moiety, and a toxin component. Saporins are type I RIPs that do not contain a cell binding domain but catalytically mediate cell death efficiently [6] once they have reached the target cell cytosol, making them a suitable candidate for immunotoxin design. Saporins also lack a domain for the efficient internalisation or transfer across the endosomal membrane. If the saporin molecule is unable to escape from the lumen of the endolysosomal compartment to reach its intracellular target, it is trapped and subsequently degraded in the lysosome [7]. One method for improving the efficacy of saporin-based immunotoxins is to increase the efficiency with which the toxin component reaches the cytosol of target cells following the endocytosis of the IT. Saponins from gypsophila plant species have been shown to augment an epidermal growth factor (EGF) targeted toxin for human carcinoma cells [8] and saporin immunotoxins directed against a variety of target molecules for carcinoma [9] and haematological cell lines [10].

Membrane vesiculation [11], the disruption of lipid domains within the membrane [12] and the formation of pores [13] are three possible mechanisms by which saponin may cause the perturbation of eukaryotic cell plasma membranes (PM). It is important to note that *Saponinum album* (SA) augments the cytotoxicity of saporin immunotoxins at non-lytic concentrations (i.e., non-plasma membrane disrupting) [4,14]. There is evidence to suggest that saponins from *Saponaria Officinalis* L. regulate the release of already internalised saporin molecules from intracellular compartments into the cytosol [4]. Fluorescence microscopy co-localisation studies have shown that the relevant intracellular compartments for enhanced cytotoxicity are late endosomes and lysosomes [4,5]. More recently, a flow cytometric method, using pulse width analysis, was effectively used to demonstrate the SA-induced endolysosomal escape of an Alexa Fluor (AF) labelled saporin immunotoxin from Daudi lymphoma cells [15]. Wensley et al. [15] showed that pulse shape analysis could be used to show the uptake of AF labelled IT into the endolysosomal compartment.

Glauert et al. [16] presented the first model of saponin action towards membranes proposing that the initial spontaneous formation of saponin cholesterol complexes in membranes was followed by the association of these complexes into ‘two-dimensional micellar-type structures’ within the membrane bilayer. Later work [11,13] led to the idea that saponins are spontaneously incorporated into the facing membrane monolayer and then assemble into 1:1 complexes with cholesterol, subsequently accumulating into matrices or plaques which alter membrane curvature. The induced curvature changes create pores or hemitubular protuberances that may eventually lead to sterol extraction via the process of vesiculation [17].

Lorent et al. [18] reported that the apoptotic and permeabilising effects of α *Hedera helix* were dependent on an interaction with membrane cholesterol which subsequently led to pore formation. Membrane toxic saponins have been shown to decrease the cholesterol content of cytoplasmic membrane fractions but significant changes in cholesterol content were not observed for saponins with little or no membrane toxicity [19]. These authors postulated that the formation of saponin cholesterol complexes would occupy more space in the membrane than an individual cholesterol molecule causing the membrane to become porous and permeable to smaller ions but to lose its fluidic character so that the integration of amphiphilic molecules like cholesterol is hindered. Smith et al. [14] investigated the effects of plasma membrane and cellular cholesterol on the augmentative and lytic properties of SA saponins towards the Daudi human lymphoma cell line. These studies showed that the SA augmentation of BU12-SAP IT cytotoxicity was cholesterol dependent through the restoration of the augmentative effect of SA when lipid deprived Daudi cells were cholesterol repleted using low density lipoprotein (LDL) [14]. The proportional levels of cholesterol vary between cell membrane type, with approximately 80% of total cellular cholesterol being found in the plasma membrane [20] and late endosomes containing half the cholesterol content of the plasma membrane [21] but the mechanism(s) of cholesterol re-distribution between intracellular membranes has not been fully elucidated. Subtil et al. [22] reported that cholesterol depletion inhibited clathrin-coated pit budding and concluded that clathrin is unable to induce curvature in cholesterol depleted membranes. It is feasible that the lower cholesterol content of endosomal membranes plays an important role in the way in which SA saponins interact with the bilayer, thus conferring the augmentative effect of SA. In the present study, we used pulse width analysis and confocal microscopy to investigate the effects of lipid depletion and cholesterol repletion on the SA-induced endosomal escape of saporin and the saporin-based immunotoxin OKT10-SAP in Daudi lymphoma cells. Further understanding of the mechanistic basis of saponin augmentation for saporin immunotoxins would potentially aid the development of saporin-based immunotoxin treatments for clinical use.

## 2. Results

### 2.1. Propidium Iodide Uptake Increases in Daudi Cells Incubated with 5 µg/mL SA for 24 H after Pre-Exposure to OKT10-SAP-AF

**Figure 1:**
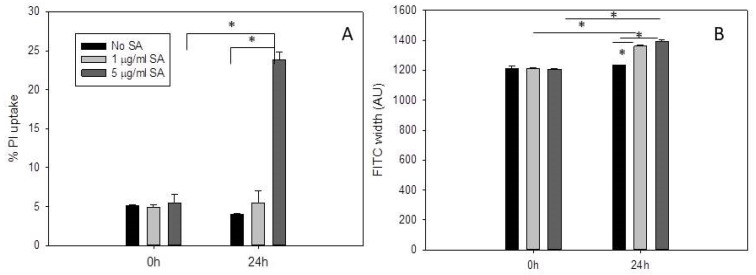
Incubation with Saponinum album at a concentration of 5 µg/mL *Saponinum album* (SA) for 24 h increases propidium iodide (PI) uptake in Daudi cells previously incubated with OKT10-SAP-AF for 24 h. (**A**) Percentage propidium uptake into Daudi lymphoma cells exposed to OKT10-SAP-AF for 24 h prior to incubation in the absence (black) or presence of 1 µg/mL SA (light grey) or 5 µg/mL SA (dark grey) for 0 and 24 h. (**B**) Fluorescein isothiocyanate (FITC) width values in arbitrary units obtained for OKT10-SAP-AF in Daudi cells in the absence (black) or presence of 1 µg/mL SA (light grey) or 5 µg/mL SA (dark grey) for 0 and 24 h. Data presented are the average of three experiments and the error bars represent one standard deviation either side of the mean. * *p* < 0.005 determined using the Mann–Whitney U test.

Pulse width analysis has been used previously to characterize the distribution of a fluorescent compound within the cell. Kang et al. [23], for example, used pulse width analysis to investigate nuclear enlargement. A fluorescence distribution that is localized and vesicular will generate a narrower pulse width compared to a diffuse cytosolic distribution which will produce a larger pulse width. See, for example, [15] where pulse width analysis was used to effectively measure the endolysosomal escape of the Alexa Fluor labelled IT, OKT10-SAP-AF, from Daudi lymphoma cells in the presence of 1 and 5 µg/mL SA. We have previously shown that saporin-based immunotoxins co-localise with LAMP1 [15] and EEA1 [14,15] indicating that these ITs accumulate in lysosomes and endosomes, respectively. In the present study, we investigated propidium iodide uptake, as a measure of membrane permeabilisation, in Daudi lymphoma cells incubated with OKT10-SAP-AF for 24 h prior to the addition of 1 µg/mL, 5 µg/mL or no SA after 0 and 24 h. We recorded fluorescein isothiocyanate (FITC) width values for the same populations of cells. Daudi cells were incubated in the presence of OKT10-SAP-AF for 24 h, washed and incubated in R10 or R10 containing 1 or 5 µg/mL SA for up to 24 h. The cells were then washed and re-suspended in propidium iodide in RPMI 1640 medium. Figure 1A shows that a concentration of 5 µg/mL SA produced a significant increase in propidium iodide uptake, from 5% to 24%, after 24 h in Daudi cells previously exposed to IT. However, Daudi cells pre-treated with IT and then exposed to a concentration of 1 µg/mL SA for 24 h did not show a significant change in propidium iodide uptake compared to the cells not exposed to SA after treatment with IT for 24 h. Daudi cells exposed to 1 or 5 µg/mL SA only for 24 h showed no increase in propidium iodide (PI) uptake (WSS unpublished results).

Concentrations of both 1 and 5 µg/mL SA produced an increase in FITC width values obtained for OKT10-SAP-AF in Daudi cells (Figure 1B). After 24 h, the FITC width value in the absence of SA was 1234 compared to 1360 in the presence of 1 µg/mL SA and 1390 in the presence of 5 µg/mL SA.

### 2.2. Lipid Depletion Reduces Endolysosomal Escape of OKT10-SAP-AF and SAP-AF Induced by the Presence of 1 and 5 µg/mL SA

**Figure 2:**
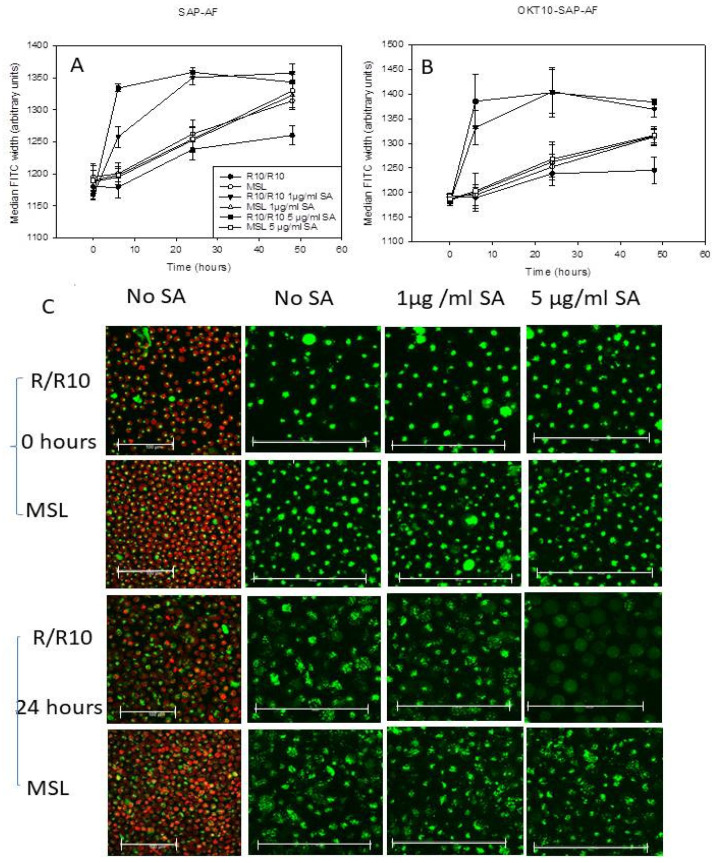
Lipid depletion of Daudi cells reduces the SA-augmented endolysosomal escape of OKT10-SAP-AF and SAP-AF. Change in FITC width for Daudi cells incubated with MβCD for 1 h followed by incubation in delipidated R10 containing lovastatin for 24 h (MSL) with SAP-AF (**A**) or OKT10-SAP-AF (**B**) for 20 h with or without (○) exposure to 1 (∆) or 5 (□) µg/mL SA compared to mock-treated Daudi cells incubated in R10 in the absence (●) or presence of 1 (▼) or 5 (■) µg/mL SA. Data presented are the mean values of three experiments and the error bars represent the standard deviation either side of the mean. (**C**) Confocal images of lipid deprived, and mock-treated control Daudi cells loaded with SAP-AF (green) for 20 h prior to the exposure to 1 or 5 µg/mL SA for zero or 24 h. Endolysosomal escape is seen as a change from a punctate compartmentalised distribution to a diffuse fluorescence throughout the cytoplasm. The images taken in the absence of SA are shown both with and without Hoechst 33342 nuclear stain (red). Images presented are the maximum projections of a series of 21 z-slices at 1 µm spacing and are representative of two independent experiments. Scale bar represents 100 µm.

Pulse width analysis was used to quantify the endolysosomal escape of Alexa Fluor 488 labelled OKT10-SAP or saporin from control or lipid depleted Daudi lymphoma cells in the presence or absence of 1 or 5 µg/mL SA. Daudi cells were lipid deprived by treatment with methyl β cyclodextrin for 1 h prior to incubation in RPMI supplemented with delipidated foetal calf serum (FCS), 2 mM glutamine and sodium pyruvate (dR10) containing lovastatin for 24 h (referred to hereafter as MSL). Four hours into the lipid deprivation treatment, the lipid deprived and mock-treated Daudi cells were exposed to Alexa Fluor 488 labelled OKT10-SAP or saporin for the remainder of the incubation period, after which the cells were washed in RPMI and re-suspended in the relevant medium, with or without SA. Figure 2A,B show the median FITC width recorded for SAP-AF and OKT10-SAP-AF, respectively, in mock-treated and lipid deprived cells exposed to 1 and 5 µg/mL SA for up to 48 h. A significant increase in median FITC width was seen in SAP-AF loaded mock-treated Daudi control cells after exposure to 1 µg/mL SA for six hours (*p* < 0.005). A concentration of 5 µg/mL SA produced a larger increase in median FITC width after six hours (*p* < 0.005). After 24 h exposure to 1 and 5 µg/mL SA, a median FITC width of 1359 arbitrary units was observed. No further increase in median FITC width was seen in the mock-treated cells exposed to SA for 48 h. Lipid deprived Daudi cells showed no significant increase in median FITC width after six hours in the presence of 1 (*p* = 0.8) or 5 µg/mL SA (*p* = 0.82). A significantly lower median FITC width value was recorded for lipid deprived cells after 24 h exposure to 1 and 5 µg/mL SA compared to mock-treated Daudi control cells, indicating the reduced endolysosomal escape of AF-SAP (*p* < 0.005). After 48 h, significantly higher median FITC width values (*p* < 0.005) were observed in lipid deprived Daudi cells in the absence of SA compared to mock-treated cells in the absence of SA indicating poor Daudi cell viability after a total of 72 h in lipid depleting conditions. The induced endosomal escape of OKT10-SAP-AF was also significantly reduced in lipid deprived Daudi cells after 6 and 24 h incubation with 1 or 5 µg/mL SA. Figure 2B shows that the median FITC width values obtained for OKT10-SAP-AF in lipid deprived cells in the presence of SA are consistent with those obtained for mock-treated Daudi control cells in the absence of SA after 6 and 24 h (Figure 2B).

Fluorescence confocal microscopy studies were also carried out on lipid deprived and mock-treated Daudi cells loaded with SAP-AF or OKT10-SAP-AF for 20 h, before the addition of SA. The distribution of SAP-AF in mock-treated cells, after 20 h incubation, was punctate in a single peri-nuclear region within intracellular compartments (Figure 2C).

Endolysosomal escape was observed in almost all Daudi control cells 24 h after the addition of 5 µg/mL SA, demonstrated by a reduction in the endolysosomal fluorescence from the labelled toxin and the appearance of fluorescence throughout the cytosol (Figure 2C). In fully supplemented cells not exposed to SA, there was an increase in the size of the discrete fluorescent regions but very few cells showed any clear cytosolic fluorescence. There was no discernible increase in the number of cells showing cytosolic fluorescence for mock-treated Daudi cells exposed to 1 µg/mL SA for 24 h compared to cells not exposed to SA. This contrasts with the increase in FITC width seen in the flow cytometry experiment and is attributed to the lower sensitivity of confocal microscopy [15]. In lipid deprived Daudi cells, the endolysosomal escape of SAP-AF was not observed 24 h after the addition of 5 µg/mL SA. Similarly, no endolysosomal escape was observed in lipid deprived Daudi cells incubated with OKT10-SAP-AF for 20 h and exposed to 1 or 5 µg/mL SA for 24 h (Supplementary Figure S1). Most mock-treated cells incubated with 5 µg/mL SA showed fluorescence throughout the cytosol, indicating the endosomal escape of OKT10-SAP-AF, after 24 h.

### 2.3. Lipid Depletion Abrogates SA Augmentation of OKT10-SAP and Saporin Using 1 and 5 µg/mL SA

**Figure 3:**
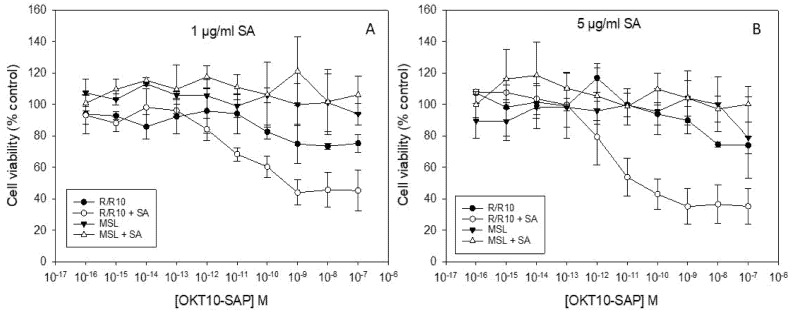
Lipid depletion of Daudi cells abrogates the SA augmentation of OKT10-SAP cytotoxicity. Dose–response curves obtained by XTT assay for lipid deprived Daudi cells in the presence (∆) or absence (▼) of 1 (**A**) or 5 (**B**) µg/mL SA exposed to increasing concentrations of OKT10-SAP compared to the mock-treated Daudi control cells in the absence (●) or presence (○) of SA. Samples were blank corrected and the absorbance at 470–650 nm was calculated for each well. The data points presented are the mean of three separate experiments and the error bars represent one standard deviation either side of the mean.

We used the XTT (2,3-bis-(2-methoxy-4-nitro-5-sulphophenyl) 2H-tetrazolium-5-carboxanilide) assay to quantify IT and saporin cytotoxicity towards lipid deprived and fully supplemented Daudi control cells after the exposure to increasing amounts of OKT10-SAP in the presence or absence of 1 and 5 µg/mL SA. Figure 3 clearly shows that the augmentation of OKT10-SAP is clearly abrogated at both 1 and 5 µg/mL SA in lipid deprived Daudi cells. We have previously shown that the augmentative effect of 1 µg/mL SA on saporin cytotoxicity was significantly abrogated in lipid deprived Daudi cells [14]. The augmentative effect of 5 µg/mL SA on saporin cytotoxicity is also completely abrogated in lipid deprived Daudi cells (Supplementary Figure S2).

### 2.4. LDL Repletion of Lipid Depleted Daudi Cells Restores OKT10-SAP-AF and AF-SAP Endolysosomal Escape Induced in the Presence of 5 µg/mL SA But Not 1 µg/mL SA

Having demonstrated that the MSL treatment of Daudi cells abolished the SA-induced endosomal escape of Alexa Fluor 488 labelled saporin and OKT10-SAP from Daudi cells (at concentrations of 1 and 5 µg/mL SA), we then investigated whether the cholesterol repletion of lipid deprived Daudi cells would restore SA-mediated endosomal escape. We used LDL to restore cholesterol to the plasma membrane. We previously showed that the incubation of lipid deprived Daudi cells with LDL repleted PM cholesterol levels back to those measured in Daudi cells incubated in full R10 [14]. In contrast, the amounts of different phospholipid molecular species determined in lipid deprived cells treated with LDL remain remarkably similar to those in lipid deprived cells and significantly different to those in untreated control cells incubated in R10 [14]. We were unable to use MβCD complexed cholesterol or Synthechol in these endosomal escape experiments because both inhibit SA activity, probably by complexing directly with SA. Our previous work produced consistent results for Daudi cell cholesterol and phospholipid composition using Synthechol or LDL for cholesterol repletion [14].

**Figure 4:**
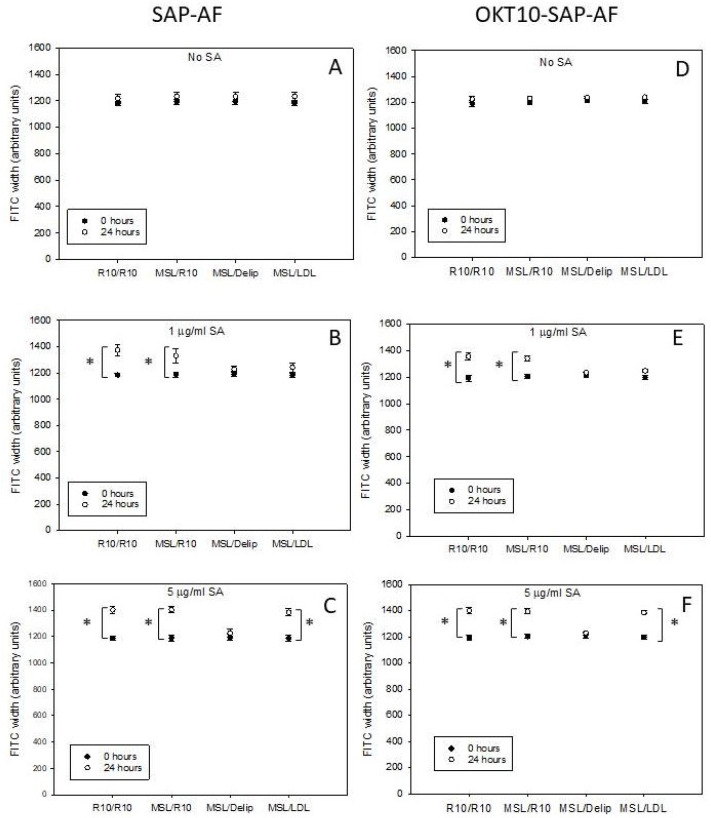
Low density lipoprotein (LDL) repletion of lipid deprived Daudi cells restores the endolysosomal escape of OKT10-SAP-AF and SAP-AF in the presence of 5 µg/mL SA. FITC width values for lipid deprived Daudi cells, lipid deprived cells repleted with LDL or R10 and mock-treated control cells after zero (black) and 24 (grey) hours for SAP-AF in the absence (**A**) or presence of 1 (**B**) and 5 (**C**) µg/mL SA and for OKT10-SAP-AF in the absence (**D**) or presence of 1 (**E**) and 5 (**F**) µg/mL SA. The data presented are the mean of three independent experiments and the error bars represent one standard deviation either side of the mean. * *p* < 0.005 as determined using the Mann–Whitney U test.

Lipid deprived Daudi cells incubated in R10 for 24 h produced median FITC width values for OKT10-SAP-AF and SAP-AF similar in magnitude to those obtained for mock-treated control cells incubated in full medium in the presence of 1 and 5 µg/mL SA, demonstrating the restoration of SA-induced endosomal escape (Figure 4). Lipid deprived Daudi cells exposed to dR10 containing LDL for 24 h showed no significant increase in OKT10-SAP-AF or SAP-AF median FITC width in the presence of 1 µg/mL SA but in the presence of 5 µg/mL SA the median FITC width values obtained were comparable to those produced by mock-treated Daudi control cells exposed to 5 µg/mL SA for 24 h. Thus, LDL restored the SA-induced the endolysosomal escape of both OKT10-SAP-AF and SAP-AF in the presence of 5 µg/mL SA but not 1 µg/mL SA. MSL-treated Daudi cells incubated for a further 24 h in dR10 showed no significant difference in the median FITC width values obtained for OKT10-SAP-AF or SAP-AF in the presence or absence of SA. The NR12S assay was used to confirm that PM cholesterol levels were repleted in lipid deprived Daudi cells after 24 h incubation in R10 or dR10 containing LDL but not in dR10 alone (Supplementary Figure S3). NR12S binds to the outer leaflet of the plasma membrane of living cells and changes its emission ratio (FIR 560/630 nm) dependent on the lipid environment [24]. During the time course (five minutes at room temperature) of this assay NR12S is not taken up into endosomal membranes and therefore provides an indication of PM cholesterol levels not endosomal membrane cholesterol levels.

**Figure 5:**
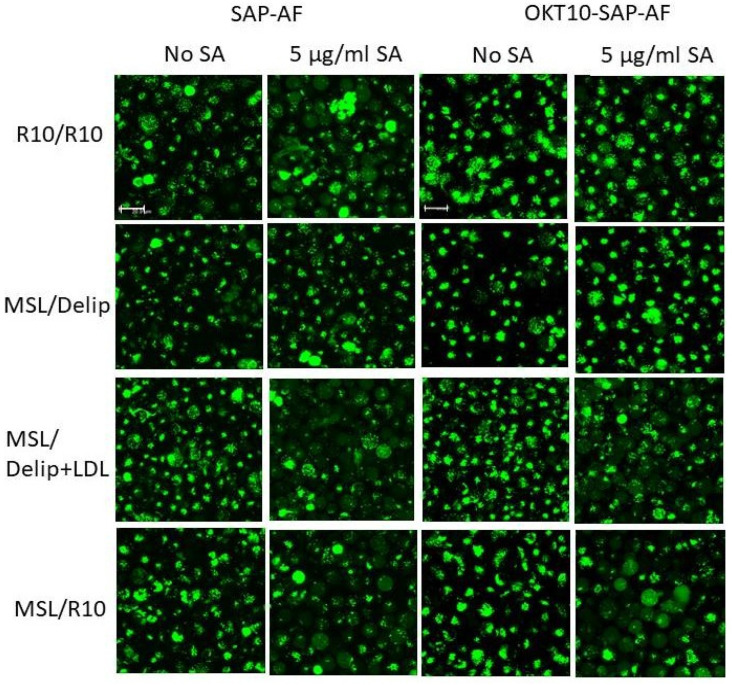
Fluorescence confocal microscopy images of mock-treated Daudi control cells, lipid deprived cells and lipid deprived cells repleted with LDL or R10 for 24 h including incubation with OKT10-SAP-AF (green) or SAP-AF (green) for 20 h prior to exposure to 5 µg/mL SA for 24 h. The images presented are the maximum projections of a series of 21 z-slices at 1 µm spacing. The scale bar represents 20 µm.

Since fluorescence confocal microscopy did not appear sensitive enough to detect endolysosomal escape of internalised SAP-AF and OKT10-SAP-AF using 1 µg/mL SA under the conditions used, we only collected images for repleted cells in the presence or absence of 5 µg/mL SA. The images presented in Figure 5 corroborate the flow cytometry results obtained for cholesterol repleted Daudi cells exposed to 5 µg/mL SA for 24 h. In the majority of lipid deprived Daudi cells incubated in R10 or dR10 containing LDL for 24 h SAP-AF, fluorescence was visible throughout the cytosol in the presence of 5 µg/mL SA, whereas in the absence of SA, very few cells showed cytosolic fluorescence. Cells exposed to 5 µg/mL SA but grown continually in lipid depleted medium produced a more intense punctate fluorescence similar to that shown by lipid deprived or mock-treated control cells in the absence of SA. The images obtained with OKT10-SAP-AF also show that endosomal escape was induced in lipid deprived Daudi cells repleted with LDL or full medium after the exposure to 5 µg/mL SA for 24 h (Figure 5). However, the SA-induced endosomal escape of OKT10-SAP-AF was not visible in the lipid deprived cells (Figure 5) after 24 h.

### 2.5. LDL Repletion of Lipid Depleted Daudi Cells Restores SA Augmentation of OKT10-SAP

**Figure 6:**
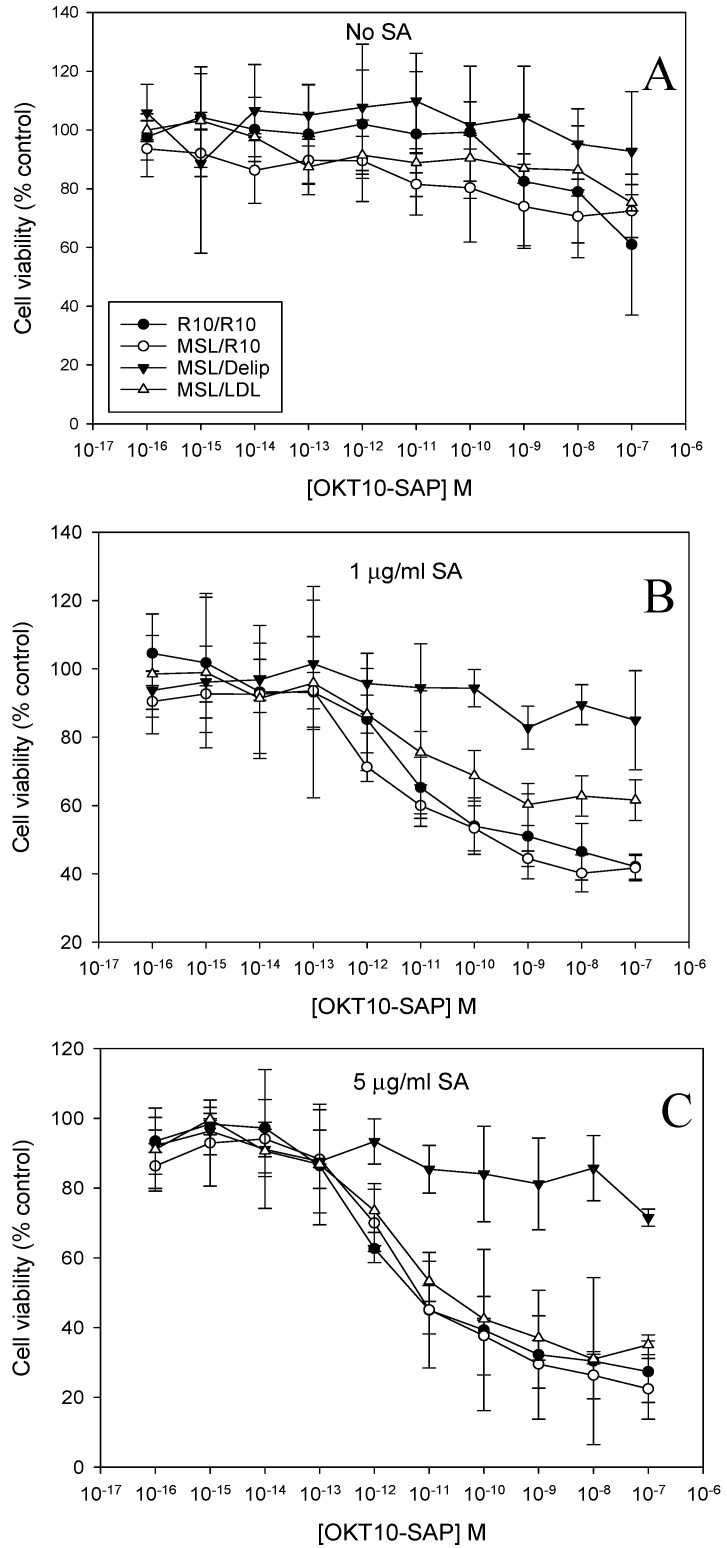
LDL repletion restores the SA augmentation of OKT10-SAP in the presence of SA. Dose–response curves obtained by XTT assay for lipid deprived cells (▼), lipid deprived cells repleted with LDL (∆) or R10 (○) compared to mock-treated control cells (●) in the absence (**A**) or presence of 1 µg/mL SA (**B**) or 5 µg/mL SA (**C**). The data points presented are the mean of three independent experiments and the error bars represent one standard deviation either side of the mean.

We then used the XTT assay to investigate whether lipid deprived Daudi cells treated with LDL, to restore their cholesterol levels, regained SA-mediated augmentation of OKT10-SAP cytotoxicity. The incubation of MSL-treated Daudi cells with LDL for 24 h restored the augmentation of OKT10-SAP by 5 µg/mL SA, as shown in Figure 6C.

Using SA at a concentration of 1 µg/mL, the almost complete restoration of the augmentation of OKT10-SAP activity was observed after the incubation of MSL-treated Daudi cells for 24 h in delipidated R10 containing LDL (Figure 6B). SA augmentation of OKT10-SAP in MSL-treated cells incubated in R10 for 24 h was completely restored using both 1 and 5 µg/mL SA. In an earlier study, we found that the augmentation of BU12-SAP cytotoxicity by 1 µg/mL SA was completely restored when lipid deprived calls were incubated in delipidated R10 containing LDL for 24 h [14].

## 3. Discussion

The purpose of this study was to determine whether the depletion of cholesterol or lipids from the cellular membranes of Daudi human lymphoma cells would also prevent the saponin-mediated release of an immunotoxin or the toxin saporin from the endolysosomal compartment to the cytosol. We were able to show that the depletion of lipids/cholesterol from Daudi cells reduced the endolysosomal escape of Alexa Fluor 488 labelled saporin and OKT10-SAP in the presence of 1 and 5 µg/mL SA. Using LDL to replete cholesterol levels, endolysosomal escape was completely restored at a concentration of 5 µg/mL SA. However, SA at 1 µg/mL failed to restore the endolysosomal escape of either OKT10-SAP-AF or SAP-AF when LDL was used to replete cholesterol levels in lipid deprived cells. The restoration of cellular lipid content using a fully supplemented medium resulted in the induction of endolysosomal escape at both SA concentrations (1 and 5 µg/mL). This discrepancy could indicate that there are different mechanisms of action for the two concentrations of SA.

Gilabert-Oriel et al. [25] determined that SA at 12 µM (~20 µg/mL) was lytic towards ECV-304 cell membranes after a 1 h incubation at 37 °C and proposed a two-stage lytic process. These authors suggested that the cell membrane may have been partially disrupted in the first phase and cells incapable of reversing this process progressed to the second phase of membrane permeabilisation. SA caused the haemolysis of 10% of red blood cells at 6 µM [25]. SA alone does not cause the immediate PM permeabilisation of Daudi cells at concentrations below 20 µg/mL SA [14], as determined by the propidium iodide (PI) uptake. However, exposure to a concentration of 5 µg/mL SA over a period of 48 h is partially cytotoxic towards Daudi cells whereas 1 µg/mL SA has no significant effect on cell viability [10]. Under the conditions used in our endolysosomal escape experiments, the PI uptake was significantly increased in Daudi cells exposed to 5 µg/mL SA for 24 h after prior incubation with OKT10-SAP-AF for 24 h, but in the presence of 1 µg/mL, the amount of PI uptake was not significantly different to that seen in Daudi cells not exposed to SA. There was no significant effect on PI uptake for Daudi cells exposed to 5 µg/mL SA with no previous exposure to OKT10-SAP-AF. These data support the hypothesis that there are possibly two different mechanisms of action involved at the two concentrations of SA. If plasma membrane permeabilisation occurs at a concentration of 5 µg/mL after the exposure to OKT10-SAP for 24 h, then it is possible that a concentration of 5 µg/mL SA under the same conditions is capable of causing endolysosomal membrane permeabilisation. An alternative explanation might be that SA complexes with free-cholesterol present in the LDL reagent counteracting the effect of SA on endolysosomal escape at 1 µg/mL in contrast to a concentration of 5 µg/mL SA which is at a sufficiently high level to overcome the inhibitory effect of free-cholesterol.

The confocal microscopy studies described here show that MSL-treated cells have smaller punctate fluorescent compartments compared with control mock-treated cells. Sobo et al. [26] found that cholesterol accumulated in the late endosomes of baby hamster kidney cells treated with 3 beta-(2-diethylaminoethoxy)-androstenone HCl (U18666) leading to an increase in compartment size. In our study, a reduction in the vesicular compartment size could possibly be due to a decrease in the amounts of cholesterol or other lipids present in the endosomal membranes. Bottger and Melzig [19] reported that membrane toxic saponins (IC_50_ < 60 uM as determined by the liberation of cellular lactate dehydrogenase) had no effect on the measurable levels of cholesterol in endosomes and lysosomes. However, the authors acknowledged that changes may not have been detectable due to the lower number of cholesterol molecules per unit membrane in late endosomes and lysosomes compared to the number in the PM [20,21]. These intracellular compartments both acquire their cholesterol through the energy-dependent receptor-mediated uptake of particles like very high density lipoprotein (VHDL) or LDL from the extracellular milieu, rather than through an undirected diffusion process [20]. This laboratory has previously demonstrated that PM cholesterol levels were restored, and total cholesterol levels replenished in lipid deprived cells incubated with LDL [14]. However, we did not investigate any changes that might have occurred to the cholesterol and lipid contents of the various intracellular vesicular compartment membranes. PM cholesterol may be necessary for the efficient uptake of saponin, but it is entirely possible that cholesterol does not play a critical role in the saponin-mediated endolysosomal escape of saporin or saporin-based ITs once present within the lumen of the intracellular compartment.

In the present study, the augmentative effect of both 1 and 5 µg/mL SA on OKT10-SAP and saporin cytotoxicity was completely abrogated in lipid deprived cells. Following the repletion of PM cholesterol, the augmentative effect of SA for OKT10-SAP was partially restored with a concentration of 1 µg/mL SA and fully restored by 5 µg/mL SA. Endosomal escape was not induced in cholesterol repleted cells by a SA concentration of 1 µg/mL. Therefore, we conclude that endosomal escape may not be the key step to the SA augmentation of saporin ITs at the lower concentration of 1 µg/mL. This conclusion is supported by the results we obtained in a previous study where we determined that nocodazole had no effect on SA augmentation when used at 1 µg/mL, whereas bafilomycin A1 abrogated SA augmentation at the same concentration [27]. Baravalle et al. [28] proposed that bafilomycin caused the inhibition of dextran transfer from early endosomes to late endocytic compartments in contrast to nocodazole which affected the dextran transfer from endosomal carrier vesicles (ECV) to late endosomes, provoking our suggestion that “the release of SA and/or saporin may occur prior to the ECV to late endosome transfer of the endocytic pathway” [27]. If this is the case, then the escape from late endosomes or lysosomes is not likely to be the mechanism responsible for the 1 µg/mL SA augmentation of saporin-based immunotoxins.

Since late endosomes and lysosomes contain a lower ratio of cholesterol than the PM, it is possible that phospholipids play a prominent role in the endosomal escape process. Lyso-bis-phosphatidic acid (LBPA), for example, was shown to be required for the release of phosphorothioate antisense oligonucleotides (PS-ASOs) from late endosomes [29] and is important for membrane deformation and fusion [30]. Goral et al. [31] suggested that the interaction of saponins with phospholipids might be equally as important as their affinity for cholesterol, based on their previous studies of the interaction of digitonin with phosphatidylethanolamine (PE) and phosphatidylserine PS [32] and Quillaja bark saponins with dipalmitoylphosphatidylcholine (DPPC) [33].

Hu et al. [34] showed that some saponins do not require the presence of cholesterol in a lipid bilayer to induce a permeability change by measuring calcein leakage from phosphatidyl choline vesicles, prepared with and without cholesterol. More recently the effect of the saponin ginsenoside Rh2 was shown to be enhanced by membrane sphingomyelin (SM) but reduced by cholesterol [35]. Membrane cholesterol depletion of human monocytic leukaemia U937 cells caused an acceleration of Rh2 cytotoxicity, whereas SM depletion delayed Rh2 cytotoxicity [36]. Sobo et al. [26] used U18666A to induce cholesterol accumulation in baby hamster kidney cells. They compared the lipid composition of control and U18666A-treated cells determining a decrease in the percentage of SM and inversion of the phosphatidylcholine (PC) to phosphatidylethanolamine (PE) ratio (1.41 in control cells, 0.7 in treated cells). Changes in the PC to PE ratio would likely alter membrane curvature since PC has a lower propensity for interfacial membrane curvature than PE (see [37]). We have previously shown that lipid deprived cells repleted with LDL retained different lipid molecular species profiles compared to those incubated in R10 including reduction in the fractions of PE18:0/20:4, PE 18:1/22:6, PE18:0/22:6, PC16:0/18:1 and phosphatidyl inositol 18:0/22:4 [14]. The size and charge of lipid headgroups and the length of the hydrocarbon chain can influence membrane curvature elastic stress [38,39]. Thus, differences in cellular membrane phospholipid composition may affect the membrane fluidity and curvature of both plasma membrane and/or intracellular vesicular compartments resulting in changes in membrane permeability. The alteration in intracellular vesicular membrane curvature that occurs due to changes in membrane lipid content may be a key factor in the mechanism of SA-induced endolysosomal escape rather than changes in the proportion of a specific lipid species or cholesterol in the cell membrane. Further research is required to determine whether other lipid species in addition to cholesterol are involved in the SA-induced endolysosomal escape of saporin immunotoxins. Determining whether membrane curvature affects the process of endolysosomal escape rather than the presence of specific lipids/cholesterol in the cell membrane could give important insights that lead to improvements in drug delivery systems dependent on disrupting endolysosomal membranes to increase drug delivery to the cytosol.

## 4. Materials and Methods

### 4.1. Cell Lines

Daudi human Burkitt lymphoma cells were obtained from the European Collection of Cell Cultures (ECACC, Porton Down, Salisbury, UK) and were authenticated using the Identifier Plus DNA profiling system (Applied Biosciences, Carlsbad, CA, USA). Cells from a working cell bank were passaged every few days, for no longer than four weeks, in RPMI 1640 medium containing 10% foetal calf serum (FCS) supplemented with 2 mM glutamine and 2 mM sodium pyruvate at 37 °C in 7% CO_2_ in a humidified environment.

### 4.2. Saponinum Album

SA was obtained from Merck (Darmstadt, Germany). This commercial preparation consists of a variety of SA species from the Gypsophila plant and has previously been analysed by Weng et al. [40]. The chemical structures for two of the most abundant saponin species, SA1641and SA1657, in the mixture, have been published elsewhere [14].

### 4.3. IT

OKT10-SAP was constructed by covalently coupling the SO6 form of saporin to OKT10 using SPDP (a heterobifunctional cross-linking reagent) as previously reported [41].The antibody:toxin ratios, calculated as a percentage of the total amount of protein present, were: 1:1; ~55%; 1:2; ~10%, 10% free antibody, 10% free saporin and 15% which could be designated either a 1:3 or a 2:2 dimer as previously shown in a gel presented in [15].

### 4.4. Fluorescent Labelling of Saporin and OKT10-Saporin

Fluorescent conjugates were constructed using Alexa Fluor 488 5-TFP (Life Technologies, Eugene, OR, USA) following the manufacturers protocol and methodology previously described in [15]. Saporin SO6 or OKT10-SAPORIN were added to 100 µL carbonate buffer (1 M NaHCO_3_, pH 9.0) and 100 µL of Alexa Fluor 488 5-TFP (10 mg/mL in DMSO) and stirred for one hour at room temperature. Unconjugated fluorophore was removed by dialysis against PBS at 4 °C. The Beer–Lambert law was used to determine the concentrations of the fluorescent conjugates by measuring the absorbance of the samples at 280 and 495 nm using a Hitachi U1100 Spectrophotometer.

### 4.5. Delipidation of Foetal Calf Serum

Foetal calf serum (Sigma Chemical Co., Poole, UK) was delipidated following the methodology described by Sprong et al. [42], filter sterilized and stored at −20 °C.

### 4.6. Cholesteterol and Lipid Depletion of Daudi Cells

Daudi cells were treated with 1 mM MβCD, to extract plasma membrane cholesterol [43,44], in non-supplemented medium (RPMI) for 1 h at 37 °C in a humidified environment of 7% CO_2_. After centrifugation and washing twice with RPMI, the pulse-treated cells were cultured in RPMI supplemented with delipidated FCS, 2 mM glutamine and sodium pyruvate (referred to as dR10), to deplete the cellular lipids and cholesterol, for 24 h at 37 °C, 7% CO_2_. The dR10 contained 1 µM lovastatin, to inhibit biosynthetic cellular cholesterol production [45]. Control cells were mock treated by incubating in RPMI for 1 h followed by continuous incubation in R10 at 37 °C, 7% CO_2_. Full details of the treatment schedules are published elsewhere [14].

### 4.7. Cholesterol Repletion

Lipid deprived or mock-treated Daudi control cells were washed twice in RPMI before being re-suspended in R10, dR10 or dR10 containing 0.016 mg/mL LDL (L3486, Thermofisher, Paisley UK) and incubated at 37 °C in 7% CO_2_ for 24 h.

### 4.8. NR12S Assay

NR12S was used to indirectly measure changes in cellular plasma membrane cholesterol levels after exposure to lipid depleting and repleting conditions, as previously described [14]. The fluorescence emission spectra of NR12S depends on the surrounding lipid order shifting towards shorter wavelengths when incorporated into a more liquid ordered phase [46], for example, a phase containing more cholesterol. A BMG Fluostar Omega plate reader (BMG Labtech, Aylesbury, UK) was used to measure the NR12S emission values at 560 and 630 nm using an excitation wavelength of 520 nm. The intensity ratio 560 nm/630 nm was then calculated. We previously validated the effectiveness of NR12S at determining the depletion of cellular PM cholesterol levels by exposing cells to increasing amounts of MβCD for 1 h, as described in [47].

### 4.9. XTT Cytotoxicity Assay

The cytotoxicity of OKT10-SAP or non-targeted saporin, used individually or in the presence of SA, was determined for quadruplicate cultures of Daudi cells using a modified version of the XTT assay reported by Scudiero et al. [48]. Cells (1 × 10^5^) were seeded into a total volume of 200 µL in the relevant medium with or without SA in 96-well plates and incubated at 37 °C, 7% CO_2_ for 24 h. The plates were read on a BMG Fluostar plate reader using a spectral scan from 300 to 650 nm.

### 4.10. Flow Cytometry

Daudi cells were incubated with 1 × 10^−6^ M SAP-AF or 5 × 10^−9^ M OKT10-SAP-AF for between 18 and 24 h. Cells were washed, by centrifugation, using RPMI and re-suspended in the relevant media at 1.25 × 10^5^ cells/well, with or without SA at 1 or 5 µg/mL in a final volume of 250 µL. The cells were incubated at 37 °C, 7% CO_2_ for up to 48 h and the cells were removed from the appropriate wells after 0, 6, 24 and 48 h. Cells were washed, re-suspended in 80 µL RPMI and the FITC-width and FITC-height of 10,000 events, for duplicate cultures of target cells, were recorded on a Cytoflex Flow Cytometer (Beckman Coulter Life Sciences, High Wycombe, UK) using Cytoflex software (Version 2.1.0.92, Beckman Coulter Life Sciences, Indianapolis, IN, USA).

Cell membrane permeabilisation was quantified by propidium uptake using flow cytometry. Daudi cells were washed in RPMI by centrifugation and re-suspended in 100 µL of 1 µg/mL propidium iodide in RPMI. Cells were identified using forward and side scatter. Appropriate gating was applied to measure the percentage of total cells with high fluorescence in each sample.

### 4.11. Confocal Microscopy

Daudi cells were incubated with 1 × 10^−6^ M SAP-AF or 5 × 10^−9^ M OKT10-SAP-AF for 18–24 h. Hoechst 33342 was added to a final concentration of 5 µg/mL thirty minutes prior to the end of the incubation time. Cells were washed, re-suspended in the relevant medium and added to 8 well glass Ibidi plates with or without SA at 1 or 5 µg/mL. The samples were incubated at 37 °C in 7% CO_2_ for 24 h. Fluorescence confocal microscopy images were acquired, after 0 and 24 h, using a Leica TCs-SP8 laser scanning confocal microscope on a DMi8 inverted microscope stand with a HC PL APO CS2 63x/1.30 glycerol immersion objective zoom 2.25 and Leica LAS-X acquisition software at 37 °C. Excitation wavelengths of 405 and 488 nm were used for Hoechst and Alexa Fluor 488, respectively.

### 4.12. Statistical Analyses

Results are presented as the mean and standard deviations of the mean. The Mann–Whitney U test was used to determine *p* values for the flow cytometry data using the IBM SPSS Statistics software.

## 5. Conclusions

Pulse width analysis was used to demonstrate that lipid depletion reduced the saponin-induced endolysosomal escape of saporin and the saporin-based immunotoxin OKT10-SAP in Daudi cells for two concentrations of saponin. Lipid depletion also abrogated the augmentative effect of SA for both saporin and OKT10-SAP cytotoxicity. The cholesterol repletion of lipid deprived Daudi cells using LDL restored the endolysosomal escape of OKT10-SAP and saporin using only the higher, partially toxic, concentration of 5 µg/mL SA. However, in the presence of LDL, the augmentative effect of SA on OKT10-SAP cytotoxicity was partially restored using 1 µg/mL SA and fully restored using 5 µg/mL SA, indicating that the augmentative effect of 1 µg/mL SA (a sub-toxic concentration) might not be dependent on endosomal escape. It is possible that two different mechanisms are operative at the two different concentrations of SA studied here. We speculate that SA-induced endolysosomal escape may be more dependent on membrane curvature than the actual amount of cholesterol present in the bilayer and consequently also affected by membrane phospholipid content.

## Supplementary Materials

Supplementary Materials can be found at https://www.mdpi.com/1422-0067/21/22/8734/s1. Figure S1: Lipid depletion of Daudi cells reduces the endolysosomal escape of OKT10-SAP-AF, Figure S2: Lipid depletion of Daudi cells abrogates SA augmentation of saporin cytotoxicity, Figure S3: Plasma membrane cholesterol levels as determined by the NR12S assay.

## Abbreviations

SA	*Saponinum album*
IT	Immunotoxin
EGF	Epidermal growth factor
AF	Alexa Fluor
SAP	Saporin
FCS	Foetal calf serum
PM	Plasma membrane
LDL	Low density lipoprotein
ECV	Endosomal carrier vesicle
RIP	Ribosomal inactivating protein
FITC	Fluorescein isothiocyanate
SM	Sphingomyelin
PC	Phosphatidylcholine
PE	Phosphatidylethanolamine
PI	Propidium iodide
LBPA	Lyso-bis-phosphatidic acid
MβCD	Methyl-beta-cyclodextrin
U18666	3 beta-(2-diethylaminoethoxy)-androstenone HCl
