# Supplementary material for: The Role of Cholesterol on Triterpenoid Saponin-Induced Endolysosomal Escape of a Saporin-Based Immunotoxin
Source: Int J Mol Sci. 2020 Nov 19;21(22):8734. doi: 10.3390/ijms21228734 (PMC7699356; doi:10.3390/ijms21228734)
Supplement: Supplementary file 1 [file ijms-21-08734-s001.pdf]

Supplementary Figures

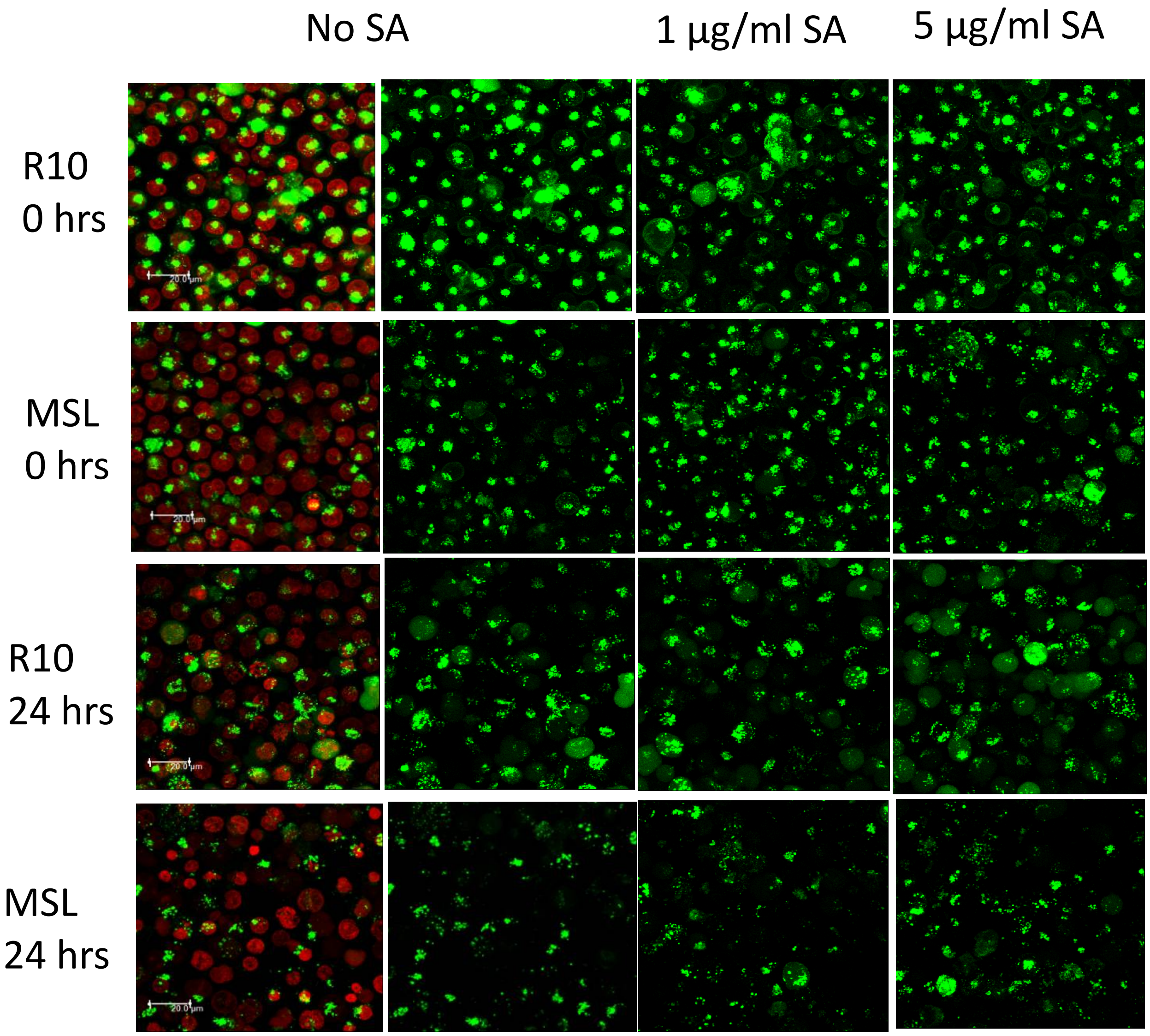

Supplementary Figure S1: Lipid depletion of Daudi cells reduces the endolysosomal escape of OKT10-SAP-AF. Confocal images of lipid deprived and mock-treated control Daudi cells loaded with OKT10-SAP-AF (green) for 20 hours prior to exposure to 1 or 5 µg/ml SA for zero or 24 hours. Images taken in the absence of SA are shown both with and without Hoechst 33342 nuclear stain (red). Images presented are maximum projections of a series of 21 z-slices at 1 µm spacing and are representative of two experiments. Scale bar represents 20 µm.

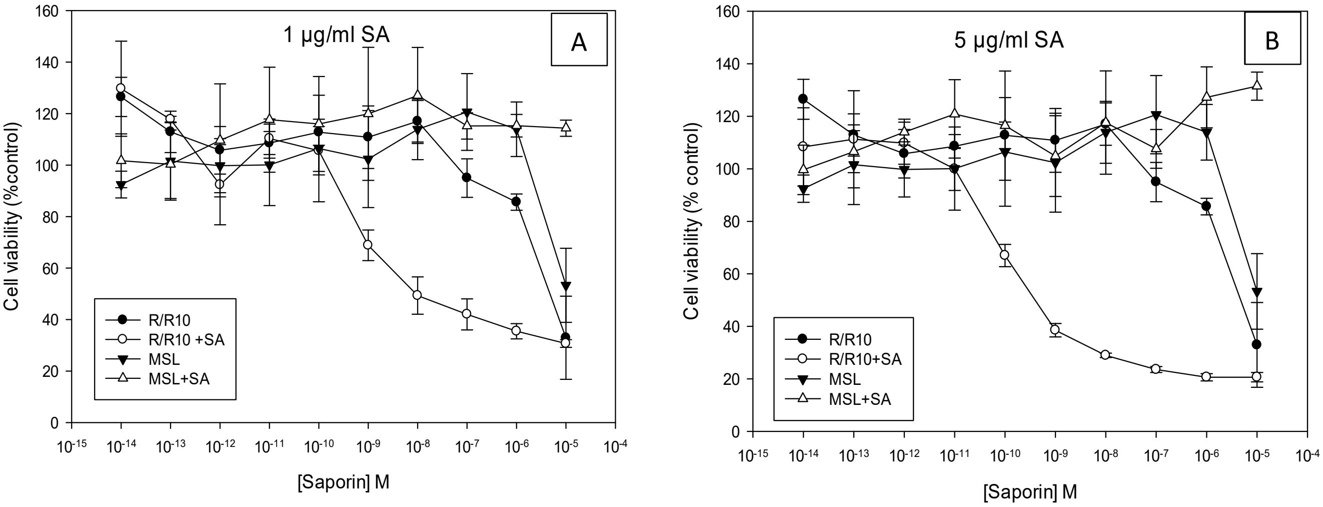

Supplementary Figure S2: Lipid depletion of Daudi cells abrogates SA augmentation of saporin cytotoxicity. Dose response curves obtained by XTT assay for lipid deprived Daudi cells in the presence (Δ) or absence (▼) of 1 (A) or 5 µg/ml SA (B) exposed to increasing concentrations of unconjugated saporin compared to mock-treated Daudi control cells in the absence (•) or presence (○) of SA . Samples were blank corrected and the absorbance at 470nm-650nm was calculated for each well. Data presented are representative of three separate experiments and error bars represent one standard deviation of the mean.

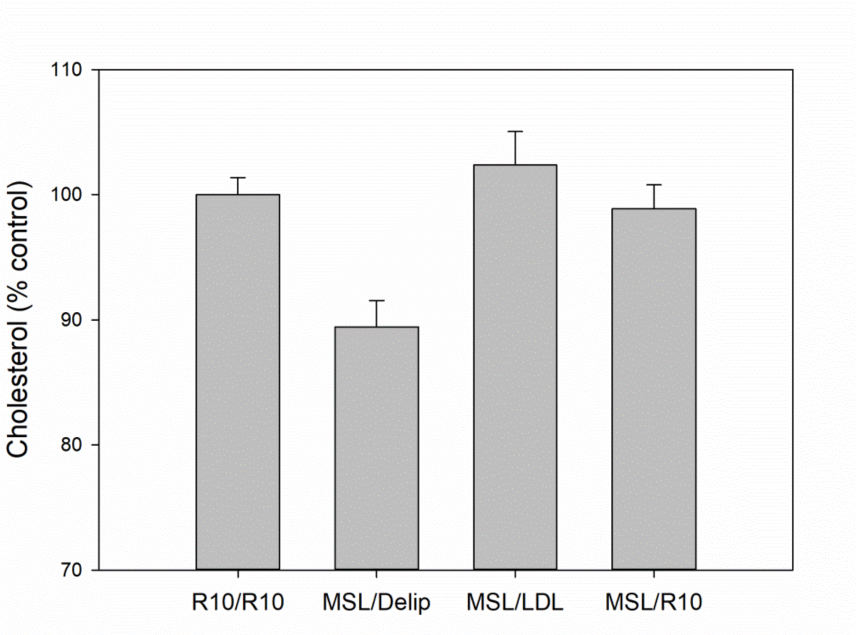

Supplementary Figure S3: Plasma membrane cholesterol levels as determined by the NR12S assay. Values are the average of quintuplicate samples and error bars represent one standard deviation either side of the mean. PM cholesterol levels were determined on the same cell samples as those used in a pulsed width analysis endosomal escape recovery experiment using OKT10-SAP-AF.
